# Mutations induced by 8-hydroxyguanine (8-oxo-7,8-dihydroguanine), a representative oxidized base, in mammalian cells

**DOI:** 10.1186/s41021-016-0051-y

**Published:** 2016-12-01

**Authors:** Tetsuya Suzuki, Hiroyuki Kamiya

**Affiliations:** Graduate School of Biomedical and Health Sciences, Hiroshima University, 1-2-3 Kasumi, Minami-ku, Hiroshima 734-8553 Japan

**Keywords:** 8-Hydroxyguanine, 8-Oxo-7,8-dihydroguanine, Specialized DNA polymerase, DNA repair protein, Nucleotide pool sanitization enzyme

## Abstract

Guanine oxidation occurs in both DNA and the cellular nucleotide pool, and one of the major products is 8-hydroxyguanine (8-oxo-7,8-dihydroguanine). The mutagenic potentials of this oxidized base have been examined in various experimental systems. In this review, we summarize the mutagenicity of the base in mammalian cells. We also describe the effects of specialized DNA polymerases, DNA repair proteins, and nucleotide pool sanitization enzymes.

## Background

DNA oxidation by reactive oxygen species has been studied for decades, due to the pivotal role of damaged DNA in processes such as mutagenesis, carcinogenesis, aging, and neurodegeneration [1–3]. Moreover, oxidized DNA precursors (2’-deoxyribonucleoside 5’-triphosphates) formed in the cellular nucleotide pool also participate in these events. Reactive oxygen species are formed endogenously and are also produced by many environmental mutagens and carcinogens. Cancer involves multiple mutations in oncogenes and tumor suppressor genes, and thus oxidized DNA and DNA precursors might contribute to an extremely high percentage of carcinogenic events.

Kasai et al. reported the formation of 8-hydroxyguanine (G^O^, 8-oxo-7,8-dihydroguanine) by the oxidation of guanine in DNA and nucleosides [4–6]. Moreover, many research groups observed its generation under various experimental conditions in vitro, in living cells, and in vivo (e.g. [7–11]). G^O^ is now recognized as one of the most important DNA lesions and has been used as a marker for DNA oxidation [12, 13], due to its prevalence in DNA and its high mutagenicity in mammalian cells. In this review, we summarize the mutagenic properties of the G^O^ damage in mammalian cells, based on results obtained in various experimental systems.

### Targeted mutations induced by G^O^

One of us (HK) constructed synthetic c-Ha-*ras* proto-oncogenes containing an G^O^:C pair in hotspots (codons 12 and 61). The modified bases were introduced into the first and second positions of codon 12 in the sense strand (5’-GGC-3’), and into the first position of codon 61 in the antisense strand (5’-CAA-3’, antisense strand 5’-TTG-3’) of the gene. Since most amino acid alterations in these hotspots activate the gene [14, 15], most base substitution mutations at the G^O^ sites induce the transformation of mouse NIH3T3 cells. Thus, the type of mutation induced by the damaged base was determined by an analysis of the gene present in focus-forming cells, after transfection into NIH3T3 cells [16, 17]. The numbers of foci formed upon the transfection of the c-Ha-*ras* genes with G^O^ were ~1 %, as compared to those formed by the activated c-Ha-*ras* genes (Val/Asp-12 and Lys/His-61).

**Fig. 1:**
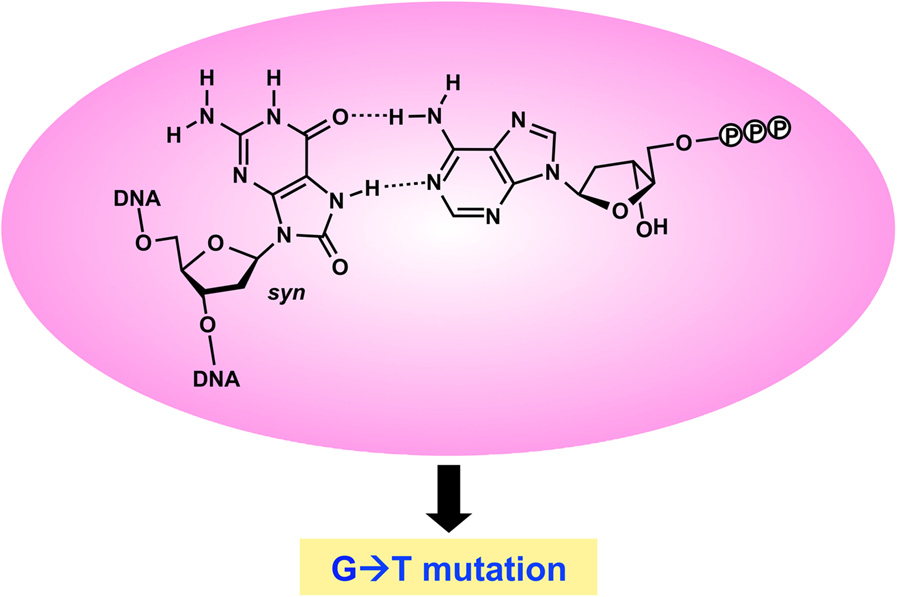
Mutation induction by dATP incorporation opposite G^O^ in template DNA. When the *syn*-oriented G^O^ base forms a Hoogsteen-type base pair with the A base of dATP in the active site of a DNA polymerase, this pairing causes the G➔T mutations.  represents a phosphate group

Sequence analysis of the c-Ha-*ras* gene present in the transformed cells indicated that the major mutation induced by G^O^ is a G➔T transversion [16, 17]. This was the first report on the mutation spectrum of this modified base in mammalian cells. This result was in good agreement with dATP incorporation opposite G^O^ by DNA polymerases (pols) in vitro [18, 19] (Fig. 1) and G^O^-induced mutations in *Escherichia coli* [20–23]. The oxidized guanine at the second position of codon 12 (5’-GG^O^C-3’) also induced a G➔A transition. The finding that the G➔A mutation was induced suggested that dTTP is also misinserted opposite G^O^ in a sequence-dependent manner, during DNA replication in NIH3T3 cells. The remarkably high thermodynamic stability (small Δ*G* ^0^ value) of the G^O^:T pair near the 5’-end (mimicking the nucleotide incorporation step) in the 5’-GG^O^C-3’ sequence may be related to the observed G➔A mutation [24]. Moreover, mutations at the 5’-adjacent positions were found, when G^O^ was incorporated into the second position of codon 12 and the first position of codon 61. Note that transformation occurs when an activated oncogene is present in the chromosomal DNA. The results observed in these studies are interpreted as the consequences of the integration of G^O^ into the chromosomal DNA and the subsequent replication of the modified chromosome(s). The relative transforming activities of the c-Ha-*ras* genes with G^O^ (~1 %) would reflect the mutation frequencies of the base in a semi-quantitative manner.

The mutagenicity of G^O^ in double-stranded (ds) plasmid DNA has been examined. Le Page et al. constructed a shuttle plasmid containing an G^O^:C pair in the sequence corresponding to codon 12 of the human c-Ha-*ras* gene (5’-GG^O^C-3’), and introduced it into simian COS-7 and human MRC5V1 cells [25]. The vector has the SV40 origin and can replicate in these cell lines. The replicated plasmid DNA was recovered and introduced into bacterial cells. The plasmid DNAs isolated from colonies were analyzed by a restriction enzyme that cleaves the plasmid without the targeted mutations and by sequencing. Among the 101 bacterial colonies analyzed, none had the mutated sequence in the COS-7 experiment. Moreover, only one colony among the 125 colonies obtained in the MRC5V1 experiment had the targeted G➔T transversion. Thus, the mutation frequency of G^O^ was less than 1 % in their experiments.

Our research group also performed similar experiments, using a shuttle vector containing the oxidized base in the *supF* gene and the SV40 origin [26–30]. The base was incorporated into the 5’-GG^O^T-3’ sequence of the gene. The G^O^ plasmid was transfected into human 293T and U2OS cells, and the replicated plasmid was introduced into the indicator bacterial cells (KS40/pOF105). Mutations in the *supF* gene are detectable using this strain, since most mutations in the gene confer nalidixic acid and streptomycin resistance to the cells. The frequencies of base substitution mutations at the G^O^ site were 3–6 and ~1 × 10^-3^ in 293T and U2OS cells, respectively. The major mutation was a G➔T transversion, but G➔A and G➔C mutations were also observed at lower frequencies. Semitargeted mutations at the 5’-flanking positions were also detected.

Sunaga et al. and Yamane et al. incorporated an G^O^:C pair into the 5’-AG^O^G-3’ sequence in the *supF* gene, and introduced the ds *supF* shuttle plasmid into NCI-H1299 cells [31, 32]. In contrast to the experiments by Le Page et al. and our research group, the G➔T mutations were induced with frequencies of 2-4 %.

Yasui et al. developed a system for the site-specific introduction of DNA lesions into human genomic DNA [33]. They used lymphoblastoid TSCER122 cells heterozygous for the thymidine kinase gene. The cells display a *TK-/-* phenotype, since one allele contains a mutation in exon 4 and the other has an I-*Sce* I site and a 356-bp deletion in exon 5. Linear 6.1-kbp DNA containing the wild-type exon 5 and an G^O^ residue (in an intron) was introduced into the cells, together with the I-*Sce* I expression DNA. The correct targeting restored the wild-type phenotype for thymidine kinase. Sequence analysis of the genomic DNA of revertant clones (*TK+/-*) indicated that the modified base induced G➔T, G➔C, and G➔A targeted mutations, with G➔T mutation frequencies of 5-8 %.

In addition to the approaches using ds DNA, the mutagenicity of G^O^ in single-stranded shuttle plasmid (phagemid) DNAs has been examined in simian cells [34–36]. G^O^ induced mutations with a frequency of 4–7 %, and the major mutation was the G➔T transversion. Moreover, G➔A and G➔C mutations were detected. Recently, Pande et al. examined the mutagenic properties of G^O^ in the 5’-TG^O^N-3’ (N = A, G, C, and T) sequence in human 293T cells [37]. The frequencies of G➔T targeted mutations were 5–11 %, and G➔A and 2-base deletion mutations were also induced. In particular, the G➔A mutation was observed as frequently as the G➔T mutation, in the case of the 5’-TG^O^G-3’ sequence. In addition to the mutations in the modified position, semi-targeted mutations at the neighboring positions were found.

### Roles of specialized DNA pols

We knocked-down DNA pols η, ι, ζ, and REV1 by their respective siRNAs in human 293T cells, and introduced the ds *supF* shuttle plasmid into the knockdown cells [26]. The knockdowns of DNA pols η and ζ enhanced the G➔T mutation by an G^O^:C pair in the plasmid, but those of pol ι and REV1 had no effect [26]. These results indicated that DNA pols η and ζ are involved in error-free bypass of the G^O^ base during the replication of ds DNA (Table 1). In contrast, the G ➔T mutation induced by the oxidized base in the ds shuttle plasmid decreased in the DNA pol κ-knockdown human U2OS cells [28]. This result suggested that DNA pol κ bypasses the G^O^ base in an error-prone manner.

**Table 1:** Expected functions of cellular proteins related to mutations by G^O^ directly produced in DNA

Protein		Role
Specialized DNA pol		
	pol η	error-free bypass
	pol ζ	error-free bypass
	pol κ	error-prone bypass
	pol λ	error-free bypass, suppression of untargeted mutations
DNA glycosylase		
	OGG1	suppression of G→T mutation by G^O^ removal
	MUTYH	suppression of G→T mutation by A removal
	NTH1	suppression of G→T mutation
	NEIL1	suppression of G→T mutation
WRN		suppression of untargeted mutations

Interestingly, the knockdown of DNA pol λ increased the 2-base deletion mutations induced by G^O^ in single-stranded DNA [37]. This result suggested that DNA pol λ is involved in the error-free bypass of G^O^, by preventing the 2-base deletion.

### Roles of DNA glycosylases

Overexpression of nuclear OGG1 or MUTYH, which are the major DNA glycosylases involved in the base excision repair of G^O^ [38], suppressed the G➔T mutation frequency in a ds *supF* shuttle plasmid containing an G^O^:C pair [31, 32]. Yasui et al. also reported that the G➔T mutation, caused by G^O^ site-specifically introduced into genomic DNA, was reduced in MUTYH-overexpressing cells [33]. In accordance with these observations, we found that the knockdowns of the OGG1 and MUTYH DNA glycosylases in human cells significantly increased the frequencies of G➔T transversion caused by G^O^:C in the *supF* shuttle plasmid [27]. Surprisingly, the G➔T mutation was also enhanced when the levels of other DNA glycosylases, NTH1 and NEIL1, were decreased. These results indicated that all of these DNA glycosylases suppress the G➔T mutations caused by G^O^:C pairs generated in DNA (Table 1).

### Untargeted mutations induced by G^O^

Our research group introduced the ds *supF* plasmid containing G^O^:C into human U2OS cells, in which the Werner syndrome protein (WRN) was knocked-down. The total *supF* mutant frequency was 1.6-fold higher in the knockdown cells, as compared to the control cells [29]. Sequence analysis indicated that the targeted G➔T mutation frequency was increased only slightly by the WRN knockdown. Instead, the knockdown promoted base substitution mutations at untargeted G (or G:C) sites with statistical significance. These “action-at-a-distance mutations” seemed to be broadly distributed throughout the *supF* gene. As discussed in the original report, there are many possible explanations for these types of mutations at this time.

Similar “action-at-a-distance mutations” were observed when the G^O^:C plasmid was transfected into cells with knocked-down DNA pol λ, one of the specialized DNA pols [30]. The untargeted mutations at the G sites were significantly increased, but the frequency of untargeted mutations at G:C pairs was not significant (*P* = 0.10). The cause(s) of the untargeted mutations remain unknown, but they could be the same as those observed in the WRN knockdown cells, since DNA pol λ interacts with WRN [39].

As mentioned above, substitution mutations were found at the 5’-adjacent positions of G^O^ [16, 17]. Interestingly, Nishimura and his colleagues found that human DNA pol η misincorporated deoxyribonucleotides opposite G at the 5’-flanking site of G^O^ in the 5’-GG^O^C-3’ sequence in vitro [40]. The 5’-flanking mutations observed in mammalian cells may involve some similar events.

### Mutations induced by 8-OH-dGTP in mammalian cells

**Fig. 2:**
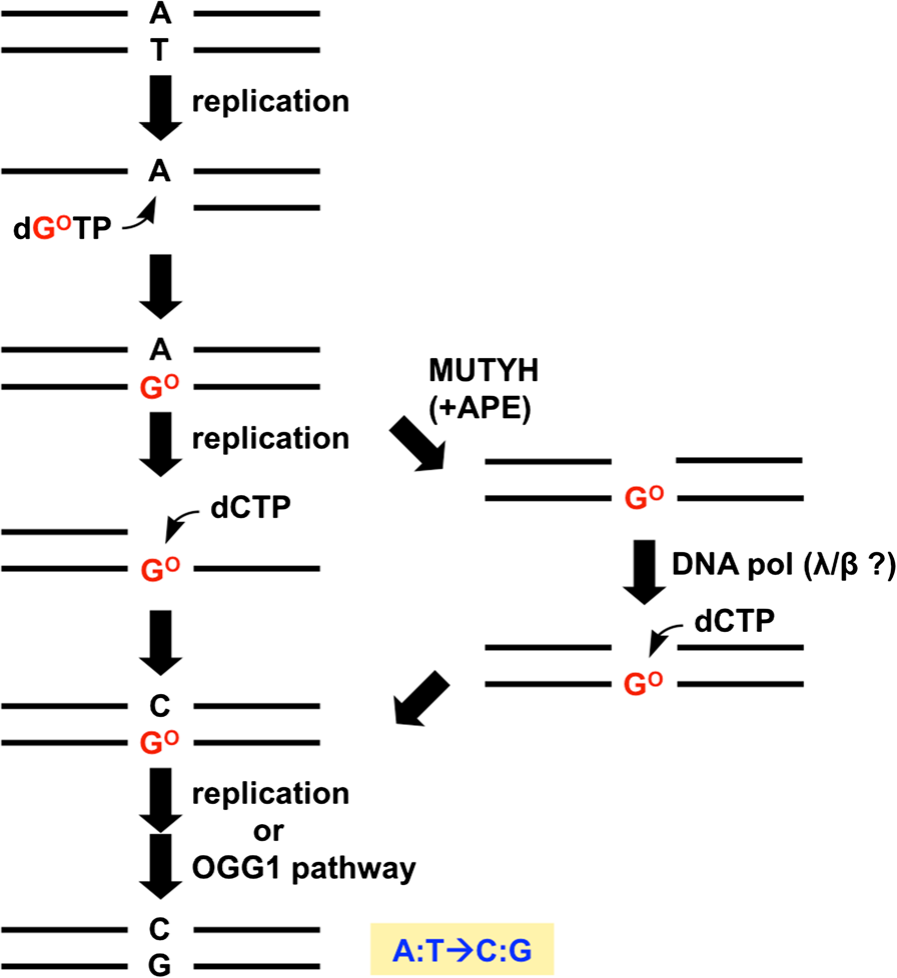
Mutagenesis pathways of dG^O^TP. APE: AP endonuclease

The ability of G^O^ to form base pairs with A and C also causes mutations when dGTP is oxidized to produce 8-hydroxy-dGTP (dG^O^TP, 8-oxo-7,8-dihydro-dGTP). Shuttle plasmid DNA containing the *supF* gene was first transfected, and then dG^O^TP was introduced into simian COS-7 and human 293T cells to examine its mutagenicity in mammalian cells. The oxidized dGTP caused A:T➔C:G transversion mutations [41, 42]. These results are consistent with observations that dG^O^TP was incorporated opposite A by DNA pols in vitro, and that the same types of substitutions were induced in *E. coli* upon treatment with the oxidized deoxyribonucleotide [43, 44]. This mutation spectrum is explained by the misincorporation of dG^O^TP opposite A, and the insertion of dCTP opposite G^O^ in DNA during the second round of replication (Fig. 2). The removal of the A bases opposite G^O^ by MUTYH and the subsequent dCTP insertion by repair DNA pols would promote the A:T➔C:G mutations induced by the incorporation of dG^O^TP (Fig. 2) (see below).

### Roles of specialized DNA pols in mutagenesis by dG^O^TP

The A:T➔C:G substitution mutations were decreased upon the knockdowns of DNA pols η and ζ, and REV1 by siRNAs in human 293T cells [42]. Thus, these specialized DNA pols seem to be involved in the mutation pathway(s) of the oxidized dGTP. To determine whether these DNA pols contribute to the incorporation of dG^O^TP and/or the insertion of dCTP opposite G^O^, plasmid DNA containing an G^O^:A pair, an intermediate in the mutagenic process of dG^O^TP, was transfected into 293T cells with knocked-down specialized DNA pols. The reduction of DNA pol η decreased the mutations induced by the G^O^:A pair by ~8 %, in agreement with the observation that dCTP is preferentially incorporated opposite G^O^ by this DNA pol [40, 45]. However, the decrease was much smaller as compared to the case of dG^O^TP-induced mutations (~32 %). Thus, the decreased A:T➔C:G mutations by dG^O^TP in the pol η-knockdown cells would be mainly due to reduced dG^O^TP incorporation into the nascent strand (Table 2). This interpretation agrees with the observation that this DNA pol incorporates dG^O^TP opposite A in a highly erroneous manner in vitro [46, 47].

**Table 2:** Expected functions of cellular proteins related to dG^O^TP-induced mutation

Protein		Role
Specialized DNA pol		
	pol η	incorporation of dG^O^TP
	pol ζ	incorporation of dG^O^TP
	REV1	incorporation of dG^O^TP
DNA glycosylase		
	MUTYH	promotion of A:T→C:G mutation
Nucleotide pool sanitization enzyme		
	MTH1	decrease of dG^O^TP
	MTH2	decrease of dG^O^TP
	NUDT5	decrease of dG^O^TP

Meanwhile, no obvious effects were observed when plasmid DNA containing G^O^:A was transfected into DNA pol ζ- and REV1-knockdown cells. Thus, the two DNA pols are likely to play important roles in the incorporation of dG^O^TP, but not in the insertion of dCTP opposite G^O^ (Table 2).

### Mutation enhancement by MUTYH DNA glycosylase

The MUTYH DNA glycosylase removes A paired with G^O^, thus preventing G➔T mutations [38]. However, this activity may promote the A:T➔C:G transversions induced by dG^O^TP. We examined this possibility by the knockdown of the enzyme and the subsequent introduction of dG^O^TP or a *supF* plasmid containing an G^O^:A pair into the cells [27]. The A:T➔C:G mutation frequency of the shuttle plasmid containing A paired with G^O^ was decreased in the MUTYH-knockdown cells. The knockdown of MUTYH also reduced the mutation frequency induced by the introduction of dG^O^TP into cells [27]. These results suggested that MUTYH promotes A:T➔C:G mutations by dG^O^TP in the nucleotide pool, although it suppresses G➔T mutations induced by G^O^ formed by the direct oxidation of DNA (Fig. 2 and Table 2).

No effects were observed when dG^O^TP or a *supF* plasmid containing G^O^:A was introduced into the cells in which the OGG1, NTH1, and NEIL1 glycosylases were knocked-down [27].

### Nucleotide pool sanitization enzymes

Nucleotide pool sanitization, the specific hydrolysis of damaged DNA precursors, is an important means by which organisms prevent mutations [48, 49]. In mammalian cells, the MTH1 (NUDT1), MTH2 (NUDT15), and NUDT5 proteins catalyze the hydrolysis of dG^O^TP and/or its diphosphate derivative to produce the monophosphate compound [50–53]. Both the *supF* plasmid DNA and dG^O^TP were introduced into cells in which the expression of each protein was knocked-down. The A:T➔C:G substitution mutations induced by dG^O^TP were higher in the knockdown cells than in control cells [54]. The increase in the induced mutation was more evident in the triple knockdown cells. These results indicated that all three proteins act as a defense against the mutagenesis induced by oxidized dGTP (Table 2).

## Conclusions

The G➔T transversion is the major targeted substitution mutation caused by the G^O^ base in mammalian cells. Moreover, the G➔A and G➔C mutations at the G^O^ site and the substitution mutations at the 5’-adjacent position of G^O^ are also induced. Action-at-a-distance mutations at untargeted positions are detected when the levels of WRN and DNA pol λ are reduced. The A:T➔C:G (A➔C) transversion is the mutation induced by dG^O^TP in the cellular nucleotide pool. The DNA repair and nucleotide pool sanitization enzymes function as the defenses against G^O^ in DNA and the nucleotide pool, respectively. MUTYH is an exceptional DNA repair protein, since it enhances the A:T➔C:G mutations when G^O^ is formed in the nucleotide pool. Some specialized DNA pols are involved in nucleotide incorporation opposite G^O^ and/or dG^O^TP incorporation, and thus affect the mutation induction by G^O^. The mutagenic properties of G^O^ are affected by various factors, including the sequence contexts and the amounts of specialized DNA pols, DNA repair proteins, and nucleotide pool sanitization enzymes. This is one of the explanations for the fact that various mutation frequencies of G^O^:C have been observed, as described above. Further studies are necessary to reveal the detailed mechanisms of the G^O^-induced mutagenesis and its suppression by cellular proteins, using various experimental systems.

## Abbreviations

dG^O^TP, 8-hydroxy-dGTP (8-oxo-7,8-dihydro-dGTP); G^O^, 8-hydroxyguanine (8-oxo-7,8-dihydroguanine); pol, polymerase; ds, double-stranded.
